# An EigenECG Network Approach Based on PCANet for Personal Identification from ECG Signal

**DOI:** 10.3390/s18114024

**Published:** 2018-11-18

**Authors:** Jae-Neung Lee, Yeong-Hyeon Byeon, Sung-Bum Pan, Keun-Chang Kwak

**Affiliations:** Department of Control and Instrumentation Engineering, Chosun University, Gwangju 501759, Korea; ljn1321@daum.net (J.-N.L.); qasdfghjt@daum.net (Y.-H.B.); sbpan@chosun.ac.kr (S.-B.P.)

**Keywords:** personal identification, principal component analysis, electrocardiogram, PCANet, EigenECG Network, CU-ECG, MIT-BIH ECG database

## Abstract

We herein propose an EigenECG Network (EECGNet) based on the principal component analysis network (PCANet) for the personal identification of electrocardiogram (ECG) from human biosignal data. The EECGNet consists of three stages. In the first stage, ECG signals are preprocessed by normalization and spike removal. The R peak points in the preprocessed ECG signals are detected. Subsequently, ECG signals are transformed into two-dimensional images to use as the input to the EECGNet. Further, we perform patch-mean removal and PCA algorithm similar to the PCANet from the transformed two-dimensional images. The second stage is almost the same as the first stage, where the mean removal and PCA process are repeatedly performed in the cascaded network. In the final stage, the binary quantization, block sliding, and histogram computation are performed. Thus, this EECGNet performs well without the use of back-propagation to obtain features from the visual content. We constructed a Chosun University (CU)-ECG database from an ECG sensor implemented by ourselves. Further, we used the well-known MIT Beth Israel Hospital (BIH) ECG database. The experimental results clearly reveal the good performance and effectiveness of the proposed method compared with conventional algorithms such as PCA, auto-encoder (AE), extreme learning machine (ELM), and ensemble extreme learning machine (EELM).

## 1. Introduction

ECG is the process of recording the electrical activity of the heart over a period of time using electrodes placed on the skin. These electrodes detect small electrical changes on the skin that arise from the heart muscle’s electrophysiological pattern of depolarizing and repolarizing during each heartbeat. In addition, The ECG signal is some kind of an electric provocation spread in the heart muscle cells. Under the influence of this provocation, the heart muscle cells shrink, which as a result, causes a mechanical effect in the form of the cyclic shrinking of heart atria and ventricles. As an effect of heart muscle shrinking, the blood circulates in the human organs. The propagation of electric provocation in the heart muscle forms a depolarization wave of the bioelectric potentials of the neighboring heart cells. The propagation of the depolarization wave is caused due to a quick movement of positive ions of sodium (Na^+^). After moving of the depolarization wave, the heart muscle cells return to their rest state recovering before starting resting negative potential. This state is called a repolarization phase. The depolarization and repolarization phenomena of the heart muscle cells are caused by the movement of ions. This is the essence of the heart electric activity. Movement of ions in the heart muscle cells is the electric current, which generates the electromagnetic field around the heart. There is a possibility to measure the electric potential at each point of the electromagnetic field [1,2]. In summary, the heart’s electrical supply is automatically functioned by slow spontaneous depolarization of the sinus node, resulting in cell migration based on relaxation and relaxation of the heart muscle. Cell migration causes depolarization and repolarization, which leads to electrical generation. Accordingly, it is the electrocardiogram that measures the cell movement of the heart from the small electricity generated by the sinus node. 

Meanwhile, ECG was originally used to distinguish diseases (arrhythmia, cardiomyopathy, and cardiac valve) in the medical field. Recently, it has been widely used in the field of recognition using characteristic information of unique ECG. Samsung pay, Apple pay, Ali pay with a fingerprint, face recognition, etc., will begin to use biometric information for personal authentication. Therefore, ECG signals that have been collected only for medical purposes can represent the person and become another unique information type. In addition, the electrocardiogram signal will have a great effect that it can be used for confirming the health status of the patient such as smart health care or telemedicine. Moreover, ECG can be used only while it is alive, and security can be useful because it cannot be authenticated if it is influenced by feelings and body changes due to intimidation. In addition, miniature devices for healthcare such as smart bands are introduced, and ECG and brain waves can be measured and utilized for personal identification at the same time [3]. The ECG of self-certification can have an effect of huge ripple as described above, and we look at trends in previous research to verify the identity of the individual.

Firstly, a detection method of heartbeat type was presented. Sotelo [4] addressed the specific problem of detecting atrial premature beats that had been demonstrated to be a marker for stroke risk or cardiac arrhythmias. Isar [5] proposed the reconstructed phase space modeled by the Gaussian mixture model and bins, separately, and subsequently used the classic Bayesian classifier for patient-independent heartbeat classification. Wen [6] applied a self-organizing cerebellar model articulation controller network to design an ECG classifier by observing the QRS complex of each heartbeat. Yu [7] presented a noise-tolerant ECG beat classification method based on the higher order statistics of sub-band components using DWT and the feedforward backpropagation neural network. 

Secondly, we discuss emotion recognition. It is one of the fields that can be developed, if ECG recognition technology is commercialized. Kiranyaz [8] presented a personalized long-term ECG classification framework, which addresses the problem within a long-term ECG signal, known as the Holter register, recorded from an individual patient. Long [9] presented a wavelet-transform-based feature extraction to recognize emotions through ECG signals. Foteini [10] studied the ECG pattern analysis for emotion detection using the Hilbert instantaneous frequency. 

Finally, we discuss biometric identification. The goal of Tantawi [11] was to quantitatively evaluate the information content of a fiducial-based feature set in terms of its effect on the subject and heart beat classification accuracy using PCA, Linear Discriminant Analysis (LDA), and information-gain ratio. Fang [12] developed a QRS detection-free ECG biometric based on the phase space trajectory of the ECG signal using normalized spatial correlation and mutual nearest point distance. David [13] proposed novel fiducial and nonfiducial approaches to electrocardiogram-based biometric systems using the mean nearest-neighbor. Singh [14] proposed new techniques to delineate P and T waves efficiently from heartbeats. Odinaka [15] proposed cardiovascular biometrics by combining mechanical and electrical signals using laser Doppler vibrometry. Juan [16] developed ECG authentication for mobile devices using ECG intervals. Safie [17] presented a novel framework comprising ECG as a biometric system for human authentication using the pulse active ratio and Euclidean distance. Juan [18] proposed using a multimodal biometric approach (ECG and fingerprint) to secure the interaction between “things” and people, and Yin [19] proposed a parallel ECG-based authentication called PEA. Specifically, this paper proposes a hybrid ECG feature extraction method that integrates fiducial- and nonfiducial-based features to extract more comprehensive ECG features and thereby improve the authentication stability. Muqing [20] presented a dynamical ECG recognition framework for human identification and cardiovascular diseases classification via a dynamical neural learning mechanism. Carmen [21] introduced a new CA scheme that, contrary to previous works in this area, considers ECG signals as continuous data streams. Pedro [22] studied the effect of attacker characterization in ECG-based continuous authentication mechanisms for the Internet of things. Mahsa [23] proposed a novel method for extracting fiducial points (FPs) of the beats in ECG signals using the switching Kalman filter. Peyman [24] developed a biometric security scheme for wireless body area networks.

In this section, well-known feature extraction methods that are employed in ECG analysis are introduced. Various feature extraction algorithms such as PCA [25], LDA [26], and Independent Component Analysis (ICA) [27] are used for personal authentication. Abawajy [25] proposed a novel multistage algorithm that combined various procedures for dimensionality reduction, consensus clustering of randomized samples, and fast supervised classification algorithms for processing high-dimensional large ECG datasets using PCA. Khalaf [26] proposed a computer-aided diagnosis system for classifying five beat types including normal, premature ventricular contraction, premature atrial contraction, left bundle branch block, and right bundle branch block using PCA with LDA. Martis [27] studied ECG beat classification using PCA, LDA, ICA, and Discrete Wavelet Transform (DWT). Also, cardiac decision is studied making using higher-order spectra using PCA [28]. Wang [29] presented a k-nearest-neighbor classifier with a heart-rate variability-feature-based transformation algorithm for driving stress recognition using LDA. Karimipour [30] introduced a simple, low-latency, and accurate algorithm for the real-time detection of P-QRS-T waves in ECG signals using DWT. 

On the other hand, there are two main types of PCANet studies. The first one is applied to various data without changing the algorithm. The second one is proposed by changing the algorithm and structure of PCANet. Researchers applying various data without changing algorithm include the following. Wu [31] utilized deep learning and SVM classifiers to recognize traffic lights for varying illumination conditions. Wang [32] showed that the error rate of PCANet is qualitatively correlated with the inverse of the logarithm of BlockEnergy, which is the energy after the block sliding process of PCANet, and also this relation is quantified by using curve fitting method.

Researchers proposing the changing algorithm of PCANet include the following. Yang [33] presented a canonical correlation analysis network (CCANet) to address image classification, in which images are represented by two-view features. Zeng [34] proposed a quaternion principal component analysis network (QPCANet), which extends PCANet by using quaternion theory, for color image classification. In addition, the first author to suggest PCANet was Chan [35]. The authors developed the PCANet for a simple deep-running algorithm. PCANet and LDANet have been developed together and compared to the existing deep-learning CNN. PCANet is similar to CNN’s convolution concept. Accordingly, there are two directions for development of PCANet. 

### Motivation and Contribution

The initial motivation of our study was to apply a simple deep learning network using ECG. PCANet is an algorithm that is primarily used in face recognition, and facial data are primarily composed of two dimensions. However, to apply ECG to EECGNet, one-dimensional ECG should be changed to 2D. How to change the dimension of the input is also seen in many trends. In addition, it is applied to many fields through recent deep running, but it is difficult to analyze the reason for its performance. That is, you cannot tell why the performance is good. Undoubtedly, EECGNet is easy to understand and analyze because it is part of the most intuitive and easy deep learning. Thus, we represent the novelty and excellence of EECGNet applications through the comparison with deep-learning algorithms in ECG.

The contribution of this paper are helping Database (DB) acquisition and performing various experiments with a preprocessing method. The preprocessing process and the data were processed. Various experiments were conducted and analyzed using EECGNet in ECG. We herein classify ECG signals through EECGNet and compare the performance with PCA, ELM and EELM. We also present a new approach using ECG signals. The ECG is originally composed of one-dimensional signals. One-dimensional ECG is featured by the proposed preprocessing method, and the feature is converted into two-dimensional. The features extracted by the PCANet using ECG are exploratory and the ECG recognition rate is improved through the following modeling. In short, the merit of this paper is the possibility of research on ECG personal recognition of research through EECGNet, and the validity of the pre-processing process, the geometric transformation of data, and algorithm are verified by using two kinds of data. In addition, the CU-ECG database is a self-produced database by the CU. 

This paper is organized as follows. Section 2 describes the concept of PCANet. The core of PCANet is PCA with learning. Hence, the Net that is called the weight is presented to train each layer, individually. Regarding speed, PCANet is slower than PCA because of the training, but PCANet performs better than PCA in terms of performance. In Section 3, we present the structure of the EigenECG Network (EECGNet), data processing, and preprocessing of ECG signals. Further, we explore the feature of EECGNet using ECG and analyze the flow of EECGNet. Section 4 describes the CU-ECG database of the construction environment and sensor information and summarizes the MIT-BIH ECG database and CU-ECG databases which are developed with a sensor to acquire ECG data. For the experiment, 1D ECG signals are transformed into 2D ones because EECGNet uses 2D and three-dimensional (3D) signals as inputs. We also use comparative indicators to analyze the differences between the data and compare their performances using PCA, Auto-Encoder (AE), ELM, and EELM. The parameters of EECGNet such as the patch size, number of filters, block size, and lap ratio are adjusted to improve its performance. We verified the influence of these parameters on the performance of PCANet by modifying the key parameters of the experiment. Finally, Section 5 presents the conclusion of this paper.

## 2. PCANet

**Figure 1:**
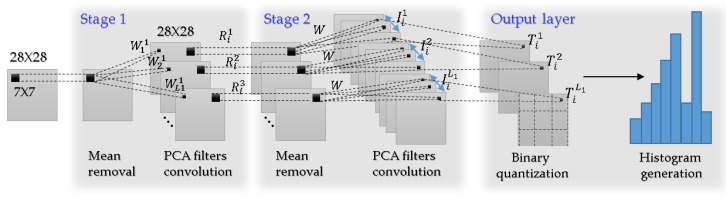
Diagram of PCANet.

In this section, PCANet-related papers and PCA papers are reviewed to apply PCANet [35]. The architecture of the basic PCANet is shown in Figure 1. It can be divided into three stages that include 10 steps. 

### 2.1. PCA

A PCA is a linear subspace projection technique used to downsample high-dimensional datasets and minimize the reprojection error. The primary steps in the process are summarized as follows. First, the covariance matrix of the input data is calculated; subsequently, from this covariance matrix, the eigenvalues and eigenvectors are calculated. Next, the eigenvalues provide a measure of the significance of the corresponding eigenvectors, and considers the variation in the original data. Finally, the eigenvectors that account for the desired level of the variation are selected. The eigenvectors are the coordinate axes of the new feature space. The variables in the original dataset are projected onto the most significant eigenvectors to yield the original data solely in terms of the chosen vectors. These transformed variables are called the principal components (PCs). The pseudocode of PCA algorithm is presented in Algorithm 1.
**Algorithm 1** PCA  **Input:** A D-dimensional training set $X=\{x_{1},\;x_{2},\ldots ,x_{N}\}$
     and the new (lower) dimensionality d (with d ≤ D)
Compute the mean $\bar{x}=\frac{1}{N}\sum_{i=1}^{N}x_{i}$Compute the covariance matrix $\mathrm{Cov}\left(x\right)=\frac{1}{N}\sum_{i=1}^{N}(x_{i}-\bar{x}){\left(x_{i}-{\bar{x}}_{i}\right)}^{T}$Find the spectral decomposition of Cov(x), obtaining the eigenvectors$V_{1},V_{2},\ldots ,V_{D}$ and their corresponding eigenvalues $\lambda_{1},\lambda_{2},\ldots ,\lambda_{D}$. Note that the eigenvalues are sorted, such that $\lambda_{1}\geq \lambda_{2}\ldots \geq \lambda_{D}\geq 0$For any $x\in {\mathbb{R}}^{D},$ its new lower dimensional representation is: $$y=\;{\left(V_{1}^{T}\left(x-\bar{x}\right),\;V_{2}^{T}\left(x-\bar{x}\right),\ldots ,V_{d}^{T}\left(x-\bar{x}\right)\right)}^{T}\in {\mathbb{R}}^{d}$$ and the original *x* can be approximated as $$x\approx \bar{x}+V_{1}^{T}\left(x-\bar{x}\right))V_{1}+(V_{2}^{T}\left(x-\bar{x}\right))V_{2}+\ldots +(V_{d}^{T}\left(x-\bar{x}\right))V_{d}$$


### 2.2. PCANet

In this section, we first review the PCANet [31,35], whose architecture is shown in Figure 1, and can be divided into three stages that include 10 steps. 

**Figure 2:**
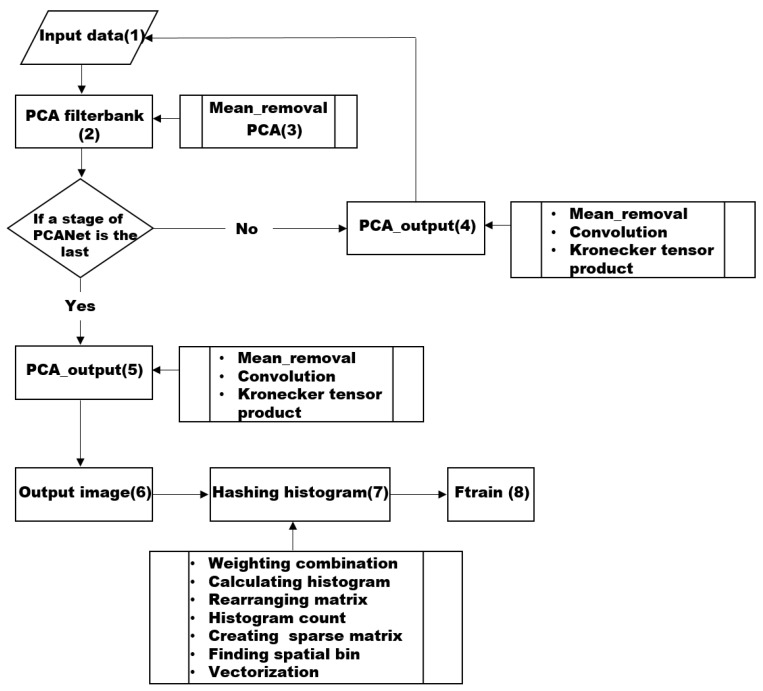
Workflow of PCANet.

Figure 2 shows the detailed flow of process that PCANet extracts features of train dataset. The detailed description of the workflow is below:

(1) The original input is training images such as ECG. However, input image is PCA output’s output if Step (4) processes before. 

(2) Extracting the PCANet filters is the main mission of PCA filterbank. 

(3) The eigenvector is an output of PCA filterbank

(4) Make original images convolve with PCANet filters as output to the next step.

(5) Make V convolve with PCANet filters as an output image to the next step.

(6) Output image is the PCANet output after two PCA stage.

(7) Binarize output image and calculate block-wise histograms. Here, we create a weight map and proceed with the binary quantization and weighted combination of the elements of the input data.

(8) Ftrain is the final feature extracted by PCANet.

Suppose that we have N input training images $\left\{I_{i},\;i=1,2,\ldots ,N\right\},\;I_{i}\in {\mathbb{R}}^{m\;\times \;n}$, and that the patch size (or 2D filter size) of all stages is $k_{1}\;$×$\;k_{2}$, where $k_{1}$ and $k_{2}$ are odd integers satisfying 1 ≤ $k_{1}$ ≤ *m*, 1 ≤ $k_{2}$ ≤ *n*. Further, the number of filters in layer i is $L_{i}$, i.e., $L_{1}$ is the first stage and $L_{2}$ is the second stage. In the following, we describe the structure of PCANet in detail. Let the *N* input images $\left\{I_{i},\;i=1,2,\ldots ,N\right\},\;$be concatenated as follows:

(1) $$\;I=\left[I_{1}\;I_{2}\;\ldots \;I_{N}\right]\in {\mathbb{R}}^{m\;\times \;Nn},\;$$

#### 2.2.1. First Stage of PCANet

As shown in Figure 1, the first stage of PCANet includes the following: 

Step 1: The first patch sliding process.

**Figure 3:**
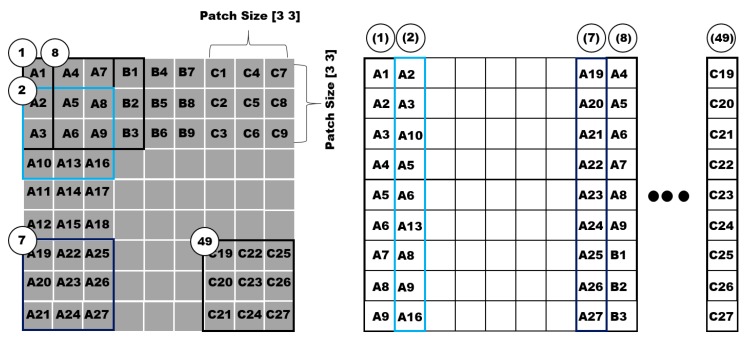
First patch sliding process.

The images are padded to $I_{i}^{\prime }\in {\mathbb{R}}^{\left(m\;+\;k_{1}\;-\;1\right)\times \left(n\;+\;k_{2\;}-\;1\right)}$ before the sliding operation. Out-of-range input pixels are assumed as zero. This ensures that all weights in the filters reach the entire images. We use a patch of size $k_{1}$ × $k_{2}$ to slide each pixel of the *i*th image $I_{i}^{\prime }\in {\mathbb{R}}^{\left(m\;+\;k_{1\;}-\;1\right)\;\times \;\left(n\;+\;k_{2}\;-\;1\right)}$, and subsequently reshape each $k_{1}\;\times \;k_{2}$ matrix into a column vector, followed by concatenation to obtain a matrix:

(2) $$\;X_{i}=\left[x_{i,1}\;x_{i,2}\;\ldots \;x_{i,mn}\right]\in {\mathbb{R}}^{k_{1}k_{2}\;\times \;mn}\;$$

where $x_{i,j}$ denotes the *j*th vectorized patch in $I_{i}.$ Therefore, for all the input training images $\{I_{i},\;i=1,\;2,\ldots ,N\},\;$we can obtain the following matrix. Figure 3 shows an example of the first patch sliding process. The data of the square matrix are vectorized according to the patch size. For example, if the patch size is [*3 × 3*], a square matrix is set as 3×3. In Figure 2, element ① (A1, A2, ..., A9) is converted to a vector. The elements are stacked from element (1) to (49) in the circle; subsequently, the mean removal step is performed using this data.

(3) $$\;X=\left[X_{1}\;X_{2}\;\ldots \;X_{N}\right]\in {\mathbb{R}}^{k_{1}k_{2}\;\times \;Nmn}\;$$

Step 2: The first mean remove process.

In this step, we subtract the patch mean from each patch and obtain the following:

(4) $$\;{\bar{X}}_{i}=\left[{\bar{X}}_{i}{,}_{1}\;{\bar{X}}_{i}{,}_{2}\;\ldots \;{\bar{X}}_{i}{,}_{mn}\right]\in {\mathbb{R}}^{k_{1}k_{2}\;\times \;mn}\;$$

where ${\bar{x}}_{i,j}\;=\;x_{i,j\;-\;1}\;-\;\frac{1}{mn}\sum_{j\;=\;1}^{mn}x_{i,j,\;}$ is a mean-removed vector. For each input training image $I_{i}\in {\mathbb{R}}^{m\;\times \;n}$, we can obtain a substituted matrix as follows:

(5) $$\;\bar{X}=\left[{\bar{X}}_{1}\;{\bar{X}}_{2}\ldots \;{\bar{X}}_{N}\right]\in {\mathbb{R}}^{k_{1}k_{2}\;\times \;Nmn}\;$$

Step 3: The first PCA process.

In this step, the eigenvalues and eigenvectors of $\bar{X}$ are calculated from Equation (5) using the PCA algorithm, which in fact minimizes the reconstruction error in the Frobenius norm as follows:

(6) $$\;\underset{V\in R^{k_{1}k_{2}\;\times \;L_{1}}}{min}\|X\;-\;VV^{T}X{\|}_{2}^{2},\;s.t.V^{T}V=I_{L1}\;$$

where $I_{L1}$ is an identity matrix of size $L_{1}\times L_{2}$, and the T denotes transposition. The PCA filters are expressed as follows:

(7) $$\;W_{l}^{1}≐\;mat_{k1}{,}_{k2}\left(q_{l}\left(XX^{T}\right)\right){\mathbb{R}}^{k1\times k_{2}},\;l=1,2,\ldots ,L1\;$$

where $mat_{k_{1},k_{2}}\left(u_{l}\right)$ is a function that maps $\left(u_{l}\right)\in {\mathbb{R}}^{k1\;\times \;k_{2}}$ to a matrix $W_{l}^{1}\in {\mathbb{R}}^{k1\times k_{2}}$. The output of the first stage of PCANet is as follows:

(8) $$\;I_{i}^{l}≐I_{i}*W_{l}^{1},\;i=1,2,\ldots ,N\;$$

where ∗ denotes a 2D convolution, and the boundary of $I_{i}$ is zero padding before convolving with $W_{l}^{1}$ such that $I_{i}^{l}$ is the same size as $I_{i}$. 

#### 2.2.2. Second Stage of PCANet

Almost repeating the same process as the first stage, as shown in Figure 1, the second stage of PCANet also includes three steps:

Step 4: The second patch sliding process. 

Similar to Step 1, we use a patch of size $k_{1}\times k_{2}$ to slide each pixel of the *i*th image $I\prime_{i,l}\in {\mathbb{R}}^{k_{1}k_{2}\times mn},\;l=1,2,\ldots ,L_{1},$ and obtain a matrix as follows:

(9) $$\;Y_{i,l}=\left[y_{i,l,1}\;y_{i,l,2}\;\ldots \;y_{i,l,mn}\right]\in {\mathbb{R}}^{k_{1}k_{2}\times mn},\;\;\;l=1,\;2,\ldots ,L_{1};i=1,2,\ldots ,N\;$$

We concatenate the matrices of all the $L_{1}$ filters and obtain the following equation:

(10) $$\;Y=\left[Y_{1},\;Y_{2},\ldots ,Y_{L1}\right]\in {\mathbb{R}}^{k_{1}k_{2}\times mn}\;$$

Step 5: The second mean removal process.

(11) $$\;\bar{Y}=\left[{\bar{Y}}_{1},\;{\bar{Y}}_{2},\ldots ,\;{\bar{Y}}_{L_{1}}\right]\in {\mathbb{R}}^{k1k2\times mnN}\;$$

Step 6: The second PCA process.

(12) $$\underset{V\in R^{k_{1}k_{2}\times L_{1}}}{min}\|\bar{Y}-VV^{T}\bar{Y}{\|}_{2}^{2},\;s.t.V^{T}V=I_{L1},\;T_{i,l}=\sum_{\ell =1}^{L_{2}}2^{\ell -1}\;$$

#### 2.2.3. Output Stage of PCANet

Step 7: Binary quantization,

In this step, we binarize the outputs $I^{II}{}_{i,l,\rho }$ of the second stage of PCANet, and obtain the following:

(13) $$\;P_{i}{,}_{l,\ell }=H\left(I_{i,l,\ell }^{II}\right),\;l=1,2,\ldots ,L1;\;\ell =1,2,\ldots ,L2;\;i=1,2,\ldots ,N,\;$$

where H(·) is a Heaviside step function whose value is 1 for positive entries, and 0 otherwise. We denote P as follows:

(14) $$P=P_{1}{,}_{1}{,}_{1}\ldots \;P_{1}{,}_{1,L_{2}}\ldots \;P_{1}{,}_{L_{1}}{,}_{1}\ldots \;P_{1}{,}_{L_{1}}{,}_{L_{2}}\ldots \;P_{N}{,}_{1}{,}_{1}\ldots \;P_{N}{,}_{1}{,}_{L_{2}}\ldots \;P_{N}{,}_{L_{1}}{,}_{1}\ldots \;P_{N}{,}_{L_{1}}{,}_{L_{2}}\;$$

Step 8: Weight and sum.

Around each pixel, we view the vector of $L_{2}$ binary bits as a decimal number. This converts the binary images $P_{i}{,}_{l,\ell }$ back into integer-valued images as follows:

(15) $$\;T_{i,l}=\overset{L_{2}}{\underset{\ell =1}{\sum }}2^{\ell -1}\;$$

We denote T as follows:

(16) $$\;T=\left[T_{1,1}\ldots \;T_{1,L_{1}}\ldots T_{N,1}\ldots T_{N,L_{1}}\right]\in {\mathbb{R}}^{m\times NLL_{1}n}\;$$

Around each pixel, we view the vector of $L_{2}$ binary bits as a decimal number. This converts the binary images $P_{i}{,}_{l,\ell }$ back into integer-valued images.

Step 9: Block sliding. 

We use a block of size $h_{1}\times h_{2}$ to slide each of the $L_{1}$ images $T_{i,l},\;l=1,\ldots ,L_{1}$, with overlap ratio *R*, and subsequently reshape each $h_{1}\times h_{2}$ matrix into a column vector, which is then concatenated to obtain a matrix as follows:

(17) $$\;Z_{i,l}=\left[z_{i,l,1}\;\;\;z_{i,l,2}\;\;\ldots \;z_{i,l,B}\right]\in {\mathbb{R}}^{h_{1}h_{2}\times B},i=1,2,\ldots ,N\;$$

where $z_{i,l,j}$ denotes the *j*th vectorized patch in $T_{i,l},\;l=1,\ldots ,\;L_{1}.\;B$ is the number of blocks when using a block of size $h_{1}\times h_{2}$ to slide each $T_{i,l},\;l=1,\ldots ,L_{1},$ with the overlap ratio *R*, and expressed as

(18) $$\;B=\left[1+\frac{m+k_{1}-1-h_{1}}{stride1}\right]\;\times \;\left[1+\frac{n+k_{2}-1-h_{2}}{stride2}\right]\;$$

where *stride*1 and *stride*2 are the vertical and horizontal steps, respectively, and round(.) means round off.

(19) $$\;stride1=round\left(\left(1-R\right)\;\times \;h_{1}\right),\;stride2=round\left(\left(1-R\right)\;\times \;h_{2}\right)\;$$

As shown in Equation (16), the number of blocks *B* increases as the overlap ratio *R* increases. For $L_{1}$ images, we concatenate $Z_{i,l}$ to obtain a matrix as follows:

(20) $$\;Z_{i}=\left[Z_{i,1}\;Z_{i,2}\;\ldots \;Z_{i,B}\right]\in {\mathbb{R}}^{h_{1}h_{2}\times L_{1}B},\;\;i=1,2,\ldots ,N\;$$

We denote Z as follows:

(21) $$\;Z=\left[Z_{1,1}\ldots \;Z_{1,B}\ldots Z_{N,1}\ldots Z_{N,B}\right]\in {\mathbb{R}}^{h_{1}h_{2}\times L_{1}BN}\;$$

Step 10: Histogram.

We compute the histogram (with $2^{L_{2}}$ bins) of the decimal values in each column of $Z_{i}$ and concatenate all the histograms into one vector and obtain the following:

(22) $$\;f_{1}={\left[Hist\left(Z_{i,1,1}\right)\ldots \;Hist\left(Z_{i,1,B}\right)\ldots \;Hist\left(Z_{i,L_{1},1}\right)\ldots \;Hist\left(Z_{i,L_{1},B}\right)\right]}^{T}\in {\mathbb{R}}^{\left(2^{L_{2}}\right)L_{1}}\;$$

This is the feature of the input image $I_{1}$ and Hist(.) denotes the histogram operation. We denote f as follows:

(23) $$\;f=\left[f_{1}\ldots f_{N}\right]\in {\mathbb{R}}^{\left(2^{L_{2}}\right)L_{1}BN}\;$$

The feature vector is subsequently sent to a classifier, for example, the Support Vector Machine (SVM) [36], etc. Figure 1 shows a diagram of PCANet. In the first stage of PCANet, mean removal and the PCA algorithm are applied. The second step is the same as the first step. Step 3 includes binary quantization and histogram computation. Table 1 shows core parameters of PCANet.

### 2.3. Comparison of PCA with EigenECGs Network (EECGNet)

The most significant difference between PCA and EECGNet is the dependency of training. EECGNet contains weights and training, while PCA is not dependent on training. Further, when PCA and EECGNet are applied as feature extractors, PCA is primarily reduced by a snapshot, but EECGNet is far from dimensional reduction. The other is the structural difference. Structurally, EECGNet contains Stage 1, Stage 2, and hashing histograms in the output layers; however, PCA contains covariance, eigenvectors, and eigenvalues. Further, these concepts are included in the EECGNet. In addition, PCA can analyze the performance according to the number of eigenvalues, but EECGNet contains various parameters (filter size, block size, patch size, and the overlap ratio of a block). Therefore, PCA compares and analyzes the performance according to the number of eigenvalues, but EECGNet can analyze the influence on various parameters and adjust the robust parameters to specific data. EECGNet is most affected by the patch size and filter size, and it is not significantly affected by the block size. The result is a large matrix formed by taking all possible products between the elements of *X* and those of *Y*.

## 3. ECG Biometrics Based on EECGNet

In this section, we present ECG-based preprocessing and EECGNet analysis. In the preprocessing, we show the sampling method by Q point detection in the original signal using ECG. The EECGNet process shows the ECG features or analyses extracted from EECGNet.

### 3.1. Preprocessing

**Figure 4:**
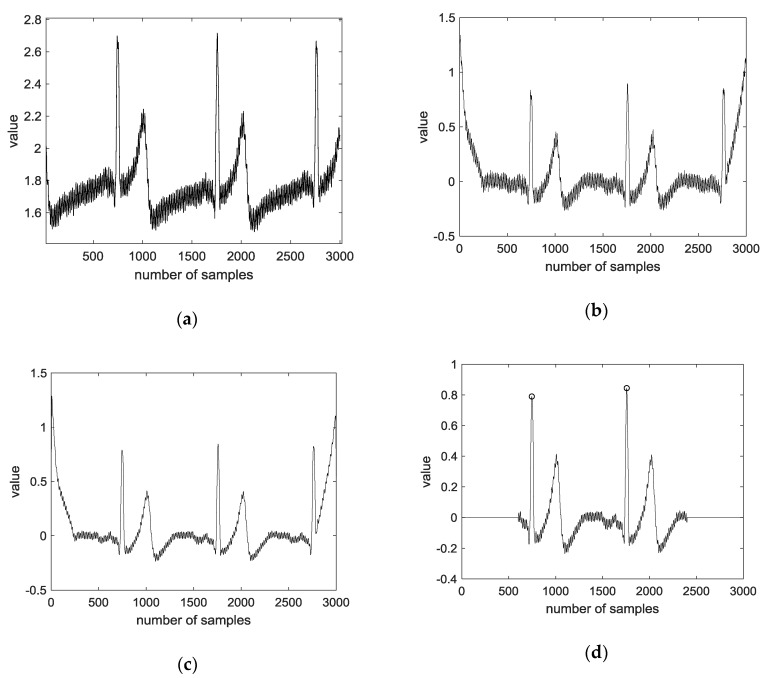
Preprocessing using CU ECG database: (**a**) raw signal; (**b**) mean variance; (**c**) spike removal; (**d**) R peak detection.

ECG recordings are typically contaminated by different types of noise and artifacts. In the preprocessing step, the goals are to reduce such noise and artifacts to determine the fiducial points (P, Q, R, S, and T), and to avoid amplitude and offset effects to compare the signals from different patients. Typical types of noise are briefly described and grouped into the following categories [37]. The preprocessing process of ECG significantly affects signal analysis and classification. Therefore, we applied the following preprocessing procedure (Steps 1–5). Figure 4 shows the preprocessing using the CU ECG database.

Step 1: Convolution is performed on the original signal with an average filter of size 500, and the average convoluted signal is subtracted from the original signal.
Step 2: Convolution is performed with an average filter of size 10 for the regular signal.
Step 3: The largest value in the signal is detected.
Step 4: An average of 400 frames are extracted based on the peaks of both sides.
Step 5: The ECG average signal of one lead and two leads are connected.

### 3.2. EECGNet-Based ECG Biometrics

**Figure 5:**
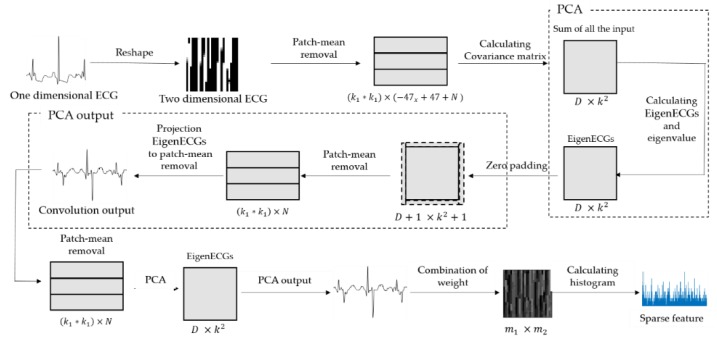
Structure of EECGNet.

**Figure 6:**
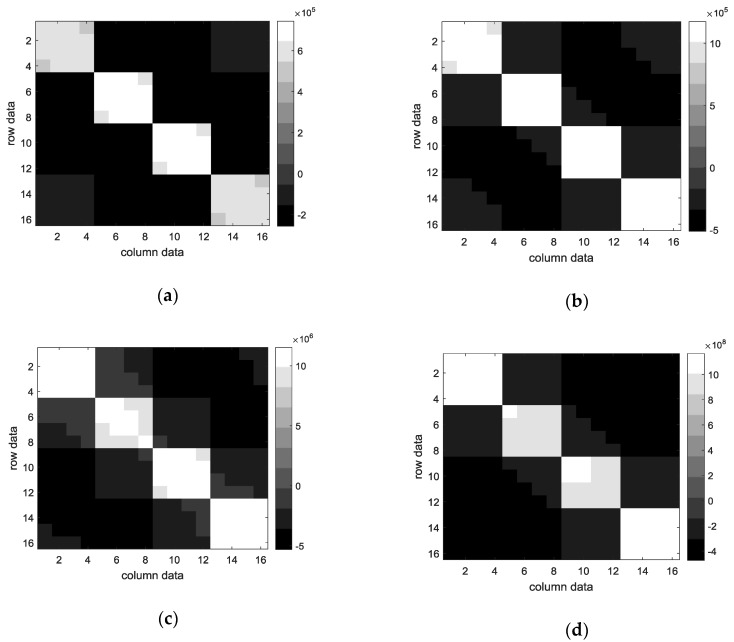
Convolution output of MIT ECG database: (**a**) first of convolution output; (**b**) second convolution output; (**c**) third convolution output; and (**d**) the last convolution output.

**Figure 7:**
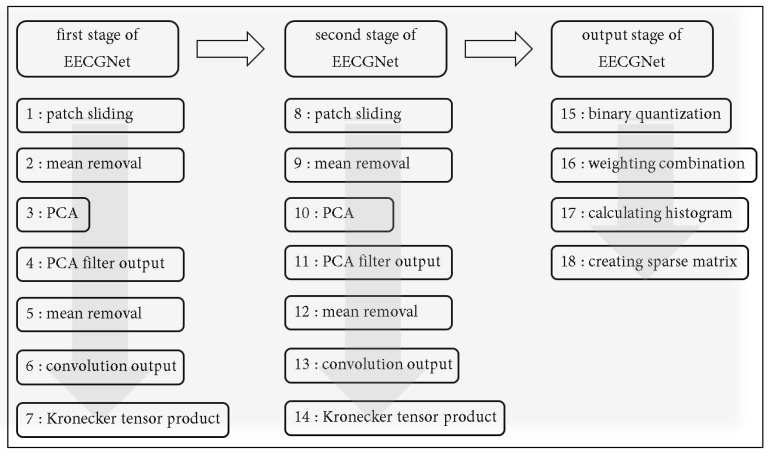
Diagram of EECGNet.

ECG authentication, which has been highlighted in the field of biometric signal authentication, is studied to design a simple and secure authentication system through a band-type clock. ECG consists of 1D data, but the inputs of EECGNet are 2D and 3D. Therefore, ECG data of size of 1 × 784 should be changed to a 2D size. We proceed with matrix resizing to transform 1D to 2D. First, we obtain a rectangular matrix of size $\left(k_{1}\;\times \;k_{1}\right)$ through patch-mean removal from 2D ECG data. Subsequently, the covariance matrix, eigenvalue, and eigenvector are obtained using PCA. This is the PCA process, and the next is the PCA output process. In the PCA output process, the size of $D+1\times k^{2}+1\;$is changed to zero by padding; subsequently, patch-mean removal is performed again. Next, the value of the patch-mean removal is projected to the eigenvector. The convolution output can be obtained at this stage. Subsequently, the rectangular matrix is obtained again through patch-mean removal and the processes of PCA and PCA output are performed again. Subsequently, when the combination of weights is performed, the size ($m_{1}\times m_{2})$ is returned to the original image size, and the sparse feature is constructed from the calculated histogram. The Kronecker is a tensor product of *X* and *Y* in the PCANet algorithm. Finally, the extracted features are used as inputs to the SVM classifier and the final recognition rate is obtained. Figure 5 shows a structure of EECGNet. Figure 6 shows convolution output of MIT ECG database. Figure 7 shows a comparison of PCA with EECGNet in structure. The pseudocode of EECGNet algorithm is presented in Algorithm 2.
**Algorithm 2** EECGNet    Input: training data ${\left\{X_{i}\right\}}_{i=1}^{N},\;X_{i}\in R^{m\times n,\;}L_{1},\;L_{2},\;The\;patch\;size\;is\;k_{1}\times k_{2}$
Reading:Signal ← ECG dataInitialization of variable:The size of the patch ← select the size of the patch in EECGNetThe number of filters ← select the number of filters in EECGNetThe size of a block ← select the size of a block in EECGNetSparsity regularization ← select the number of layers in EECGNetStage 1 of EECGNet
-Patch mean-removal:$\;X=\left[{\bar{X}}_{1},\;{\bar{X}}_{2},\ldots ,\;{\bar{X}}_{N}\right]\in R^{k1k2\times mnN}\;$-Compute convolution kernels use PCA:$$\underset{V\in R^{k_{1}k_{2}\times L_{1}}}{min}||X-VV^{T}X{||}_{2}^{2},\;s.t.V^{T}V=I_{L1}$$
$$W_{l}^{1}≐\;mat_{k1}{,}_{k2}\left(q_{l}\left(XX^{T}\right)\right){\mathbb{R}}^{k1\times k_{2}},\;l=1,2,\ldots ,L1$$
-Output: $I_{i}^{l}≐I_{i}*W_{l}^{1},\;i=1,2,\ldots ,N$Stage 2 of EECGNet
-Patch mean-removal: $\;\bar{Y}=\left[{\bar{Y}}_{1},\;{\bar{Y}}_{2},\ldots ,\;{\bar{Y}}_{L_{1}}\right]\in {\mathbb{R}}^{k1k2\times mnN}\;$-Compute convolution kernels use PCA:$$\underset{V\in R^{k_{1}k_{2}\times L_{1}}}{min}||\bar{Y}-VV^{T}\bar{Y}{||}_{2}^{2},\;s.t.V^{T}V=I_{L1}$$$$W_{\vartheta }^{2}≐\;mat_{k1}{,}_{k2}\left(q_{l}\left(XX^{T}\right)\right){\mathbb{R}}^{k1\times k_{2}},\;\vartheta =1,2,\ldots ,L2$$-Output: $O_{i}^{l}≐{\left\{I_{i}^{l}*W_{\vartheta }^{2}\right\}}_{\vartheta =1}^{L2}$Output layer
-Binary hashing: compute the decimal-valued image$$I_{i}^{l}≐\sum_{\vartheta =1}^{L_{2}}2^{\;\vartheta -1\;}H\left(I_{i}^{l}*W_{\vartheta }^{2}\right)$$-Histogram: $f_{i}≐{\left[Bhist\left(I_{i}^{1}\right),\ldots ,\;Bhist\left(I_{i}^{L1}\right)\right]}^{T}\in {\mathbb{R}}^{2^{L2})L_{1}B}$-Output: $F=\left[f_{1},\;f_{2},\ldots ,f_{N}\right]$


## 4. Experimental Results

This section lists the database acquisition process and environment, evaluates the data, and examines the similarities. In addition, we present the performance evaluation and the effectiveness of the EECGNet.

### 4.1. ECG

**Figure 8:**
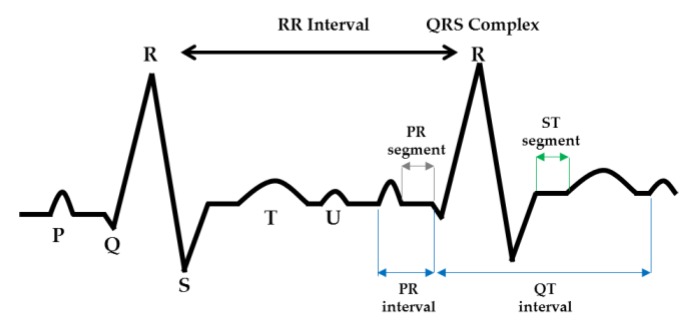
The ECG waves, segment, and intervals.

The ECG measurement method induces three types of induction: one lead (right hand, left hand), two leads (right hand, left foot), and three leads (left hand, left foot). In this paper, we use the simplest method, which is one lead, and the signal appears as PQRST wave due to contraction of the ventricle. The P-wave is the first deflection of the ECG. It results from depolarization of the atria. Atrial repolarization occurs during ventricular depolarization and is obscured. The QRS complex corresponds to the ventricular depolarization. The T-wave represents ventricular repolarization, restoration of the resting membrane potential. In about one-quarter of population, a U-wave can be seen after the T-wave. This usually has the same polarity as the preceding T-wave. It has been suggested that the U-wave is caused by after-potentials that are probably generated by mechanical–electric feedback. Inverted U-waves can appear in the presence of left ventricular hypertrophy or ischemia. The PQ segment corresponds to electrical impulses transmitted through the S-A node, bundle of His and its branches, and the Purkinje fibers and is usually isoelectric. The PQ interval expresses the time elapsed from atrial depolarization to the onset of ventricular depolarization. The ST-T interval coincides with the slow and rapid repolarization of ventricular muscle. The QT interval corresponds to the duration of the ventricular action potential and repolarization. Then, TP interval is the period for which the atria and ventricles are in diastole. The RR interval represents one cardiac cycle and is used to calculate the heart rate [1,2,38]. Figure 8 shows the ECG waves, segment, and intervals.

### 4.2. CU-ECG Database

**Figure 9:**
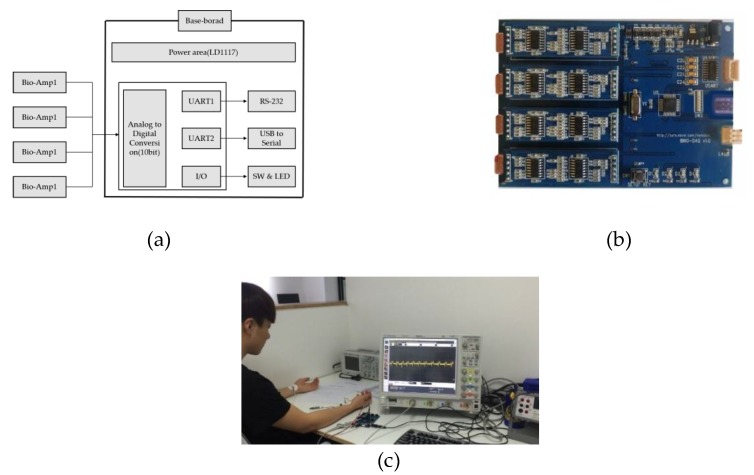
Device of CU-ECG database and internal block diagram: (**a**) block diagram; (**b**) primary board; (**c**) experiment environment; and (**d**) testing data of CU-ECG database.

The CU-ECG is the data from the CU in Korea. The data consist of 100 persons, the measurement time was 10 s for one measurement, and a total of 60 times was measured during three days. The participants were measured while they were sitting on a chair in a relaxed state, and the data sampling rate was 500,000 Hz. The type of ECG acquired was only Lead1, and the type of electrode was a wet electrode. We used a processor, amplifier, bandpass filter, band stop filter, and low-pass filter for the primary board and sensor. The processor uses Atmega8, and the Analog to Digital (AD) converter uses a 10-bit resolution. The communication is USB to serial. The gain of the amplifier was 1000 times, and the signal was measured using a 5-V positive power source. Figure 9 shows the internal block diagram of the ECG measuring device.

**Figure 10:**
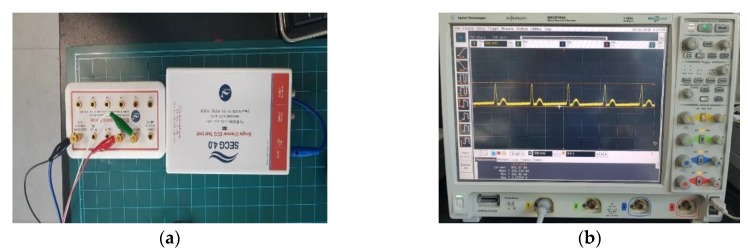
ECG performance tester for compliance tests with signal: (**a**) SECG 4.0; and (**b**) the signal extracted by SECG 4.0 using oscilloscope.

To determine the suitability of the sensor, we compared the input and output signals using an SECG 4.0 machine which is manufactured in Gyeonggi, South Korea [39]. In addition, it was confirmed that the signal was outputted according to Association for the Advancement of Medical Instrumentation (AAMI) EC11 standard. The ECG performance tester designed for compliance tests. The SECG 4.0 is compliant with IEC 60601-2-25, IEC 60601-2-27, IEC 60601-2-47, and AAMI EC11 [39]. The experiment sequence is to send a signal from the computer to the generator and connect the toolbox to the mainboard ECG system. Next, an output port is created in the system and measured with an oscilloscope. Figure 10 shows ECG performance tester for compliance tests with signal. 

### 4.3. MIT-BIH ECG Database

**Figure 11:**
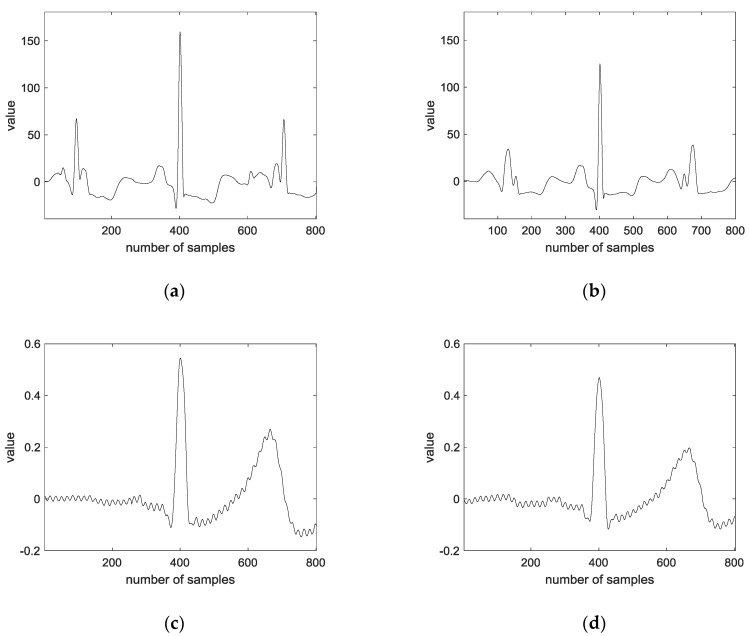
Training and testing feature data of 1D ECG using preprocessing: (**a**) training data of MIT-BIH ECG database; (**b**) testing data of MIT-BIH ECG database; (**c**) training data of CU-ECG database; and (**d**) testing data of CU-ECG database.

The MIT-BIH ECG database includes 48 parts that contain two-channel ECG recordings. Those parts were recorded between 1975 and 1979 at Boston’s Beth Israel Hospital (now the Beth Israel Deaconess Medical Center). The MIT-BIH ECG database was obtained from 47 persons, including 25 men and 22 women. Further, the men were 32–89 years of age, and the women were 23–89 years of age (two records are collected from the same male participant among all the records). This database was acquired in approximately 30 min. The sampling rate is 360 samples per second, and the resolution for digitization is 11-bit over a 10 mV range. Twenty data are constructed per class. The size of the training data is 940 × 1600, and both one lead and two leads are used. Figure 11 shows the training and testing feature data of 1D ECG using preprocessing.

### 4.4. Data Evaluation and Similarity Measurement

The compression ratio (CR) is defined as the ratio of the original signal size to the compressed signal size. The CR provides information about the degree by which the compression algorithm removes the redundant data. A higher CR requires fewer bits to store or transmit the data, which can be defined as

(24) $$\;CR=B_{0}/B_{c}\;$$

where $B_{0}$ is the total number of bits required to represent the original data, and $B_{c}$ is the total number of bits required to represent the compressed data. The percent mean square difference (*PRD*) measures the error between the original and reconstructed signals.

(25) $$\;PRD\left(\%\right)=100\;\times \sqrt{\overset{N}{\underset{n=1}{\sum }}\frac{{\left(X_{s}\left(n\right)-X_{r}\left(n\right)\right)}^{2}}{(X_{s}{\left(n)\right)}^{2}}}\;$$

The percentage root mean square difference normalized (*PRDN*) is a normalized version of the PRD, which is independent of the signal mean value $\bar{X}$.

(26) $$\;PRDN\left(\%\right)=100\;\times \sqrt{\overset{N}{\underset{n=1}{\sum }}\frac{{\left(X_{s}\left(n\right)-X_{r}\left(n\right)\right)}^{2}}{(X_{s}{\left(n)-\bar{X}\right)}^{2}}}\;$$

The root mean square error (*RMS*) provides a measure of error in the reconstructed signal with respect to the original signal.

(27) $$\;RMS\left(\%\right)=100\;\times \sqrt{\frac{\sum_{n=1}^{N}{\left(X_{s}\left(n\right)-X_{r}\left(n\right)\right)}^{2}}{\left(N-1\right)}}\;$$

The signal-to-noise ratio (*SNR*) is the measure of the degree of noise energy introduced by compression in decibels (dB).

(28) $$\;SNR=10\;\times log\left[\overset{N}{\underset{n=1}{\sum }}\frac{{\left(X_{s}\left(n\right)-\bar{X}\right)}^{2}}{{\left(X_{s}\left(n\right)-X_{r}\left(n\right)\right)}^{2})}\right]\;$$

**Figure 12:**
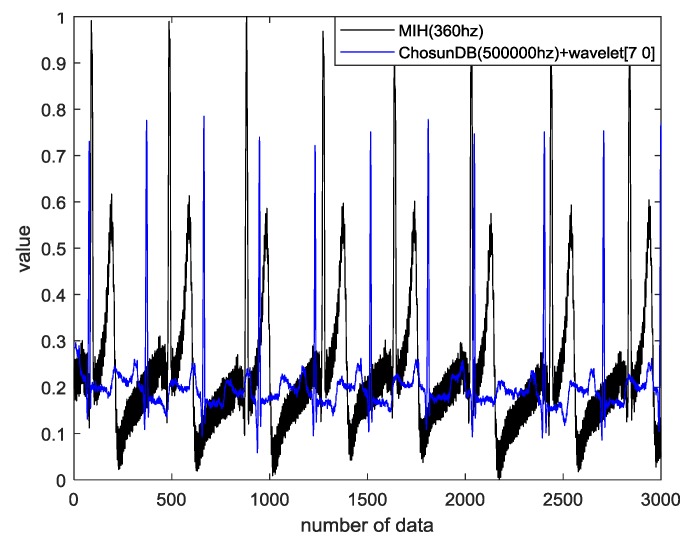
Comparison of MIT-BIH ECG database with wavelet decomposition and CU-ECG database without wavelet decomposition (*SNR* = −2.3, *MSE* = 0.0, *PRD* = 83.0, and *RMSE* = 0.2).

Figure 12 shows the comparison of the MIT-BIH ECG database with wavelet decomposition and the CU-ECG database without wavelet decomposition.

### 4.5. Performance Evaluation

The accuracy performance of the SVM is used as the ratio of the correct classification to the number of total classified samples. The accuracy can be formulized as follows:

(29) $$\;Accuracy=\frac{TP+TN}{TP+TN+FP+FN}$$

**Figure 13:**
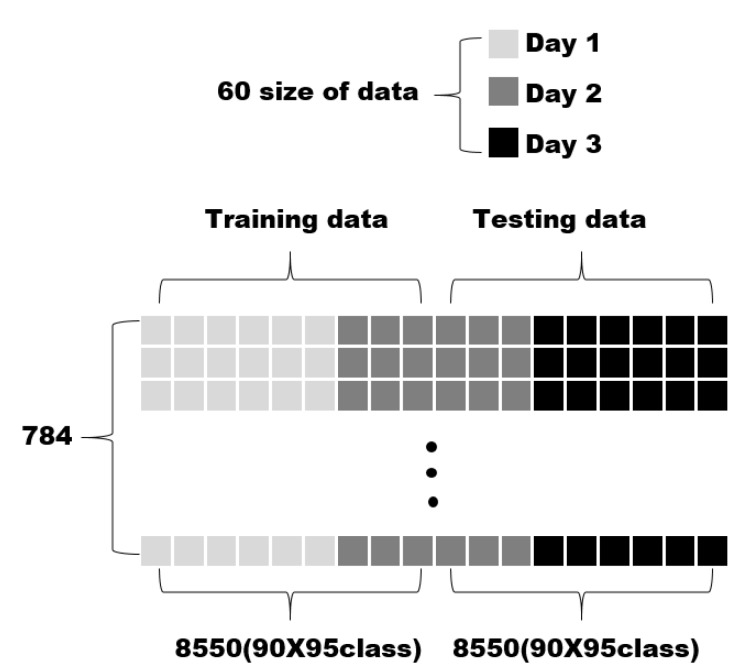
How to divide training data and testing data using CU-ECG database.

TP is the number of correct predictions for the positive samples, TN is the number of correct predictions for the negative samples, FN is the number of incorrect predictions for the positive samples, and FP is the number of incorrect predictions for the negative samples. Next, to extract the optimal performance of the EECGNet, various parameters were changed. The modified parameters correspond to *h*, *k*, *l*, and *R*. We set each parameter as $h_{1}=h_{2},\;k_{1}=k_{2},\;L_{1}=L_{2}$ and extracted the performance accordingly. We adjusted each parameter for the performance of the experiment and analyzed the effects of the parameters. The analysis shows that the higher is the block size, the better is the recognition rate. The filter number generally shows a good performance between 4 and 8. Next, concerning the database, we performed ECG personal authentication using CEECGNet. The size of both the training data and the testing data is 784 × 8550 on the CU-ECG database. There are 17,100 sizes, with 180 ECG signals per class, consisting of 95 classes. The training and testing data are divided by 0.5, resulting in a size of 8550. Training and testing data were selected as shown in Figure 13. Figure 13 shows how to divide training and testing data.

**Figure 14:**
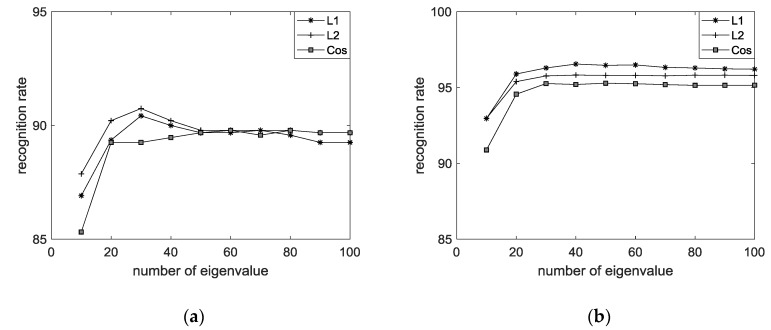
Performance of PCA using MIT-BIH ECG database and CU-ECG database: (**a**) MIT-BIH ECG database with PCA; and (**b**) CU-ECG database with PCA.

**Figure 15:**
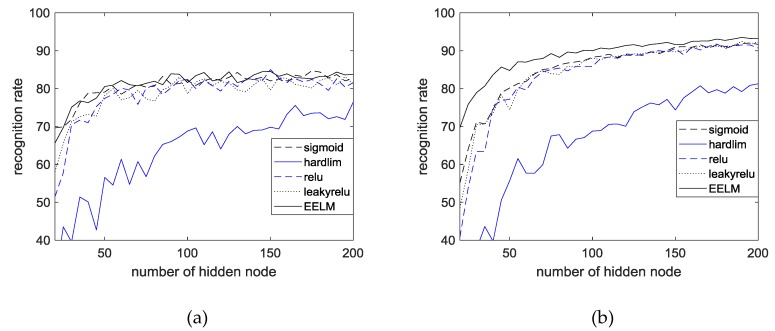
Performance of ELM using MIT-BIH ECG database and CU-ECG database: (**a**) MIT-BIH ECG database; and (**b**) CU-ECG database.

**Figure 16:**
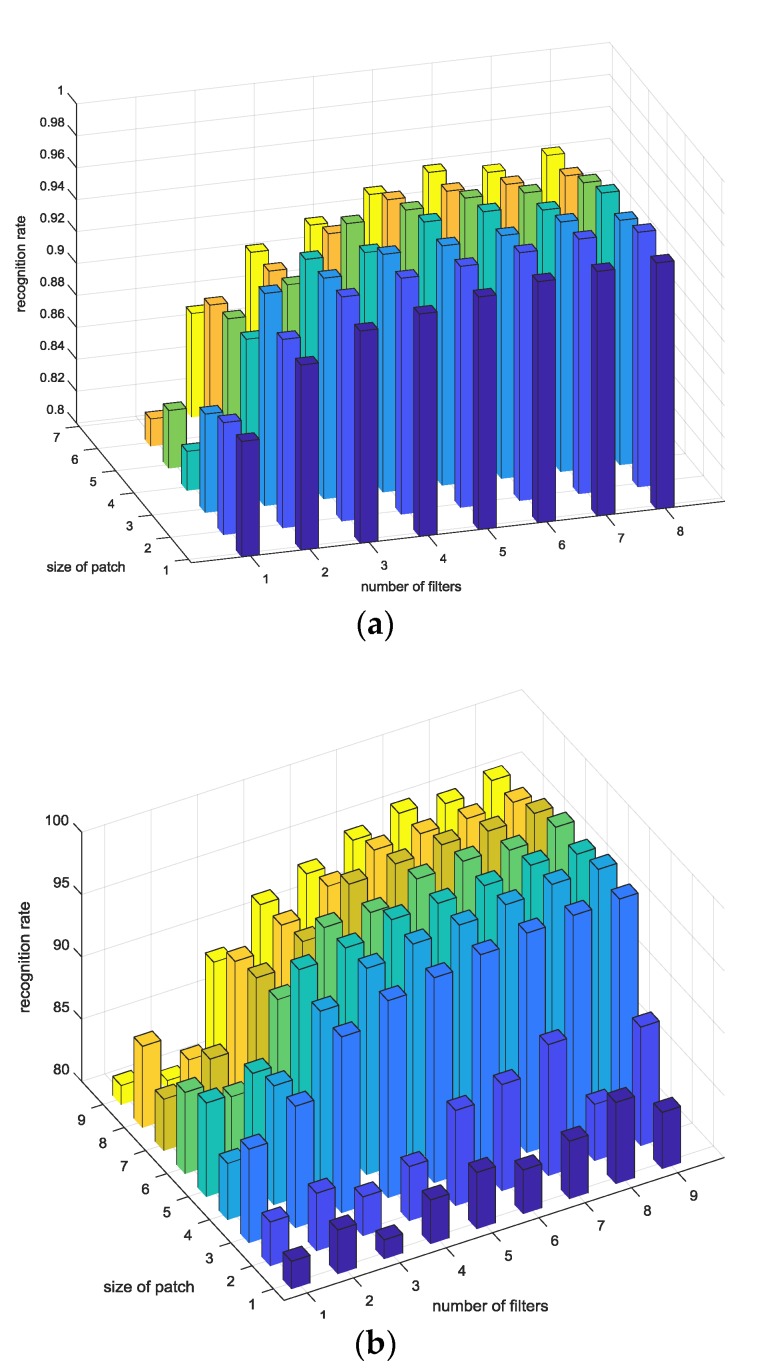
Performance for MIT-BIH ECG database using only one lead: (**a**) MIT-BIH ECG database; and (**b**) CU-ECG database.

Table 2 shows the performance of EECGNet using the CU-ECG database. Table 3 shows the performance of EECGNet using the MIT-BIH ECG database. Table 4 shows a comparison among EECGNet, PCA, AE, ELM, and EELM. Figure 14 shows the performance of the MIT-BIH ECG database using one lead. Figure 15 shows the performance of ELM using the MIT-BIH ECG database and CU-ECG database. Figure 16 shows the identification performance for the MIT-BIH ECG database using one lead.

### 4.6. Results and Discussion

We recruited 100 subjects and and acquired three days of data per person. We propose an EigenECG Network (EECGNet) based on the principal component analysis network (PCANet) for the personal identification of electrocardiogram (ECG) from human biosignal data. In this paper, we propose the classification of ECG for identification systems using EECGNet from the human biosignal data. ECG authentication, which has been highlighted in the field of biometric signal authentication, was studied to design a simple and secure authentication system through a band-type clock. We designed the EECGNet-based SVM classifier for the ECG authentication system. The design results show good performance when compared with other algorithms, and the validity of EECGNet was confirmed. The validity of EECGNet was confirmed to be 98.2% and the proposed method showed good performance when compared with conventional algorithms such as PCA, auto-encoder, ELM, and EELM.

**Figure 17:**
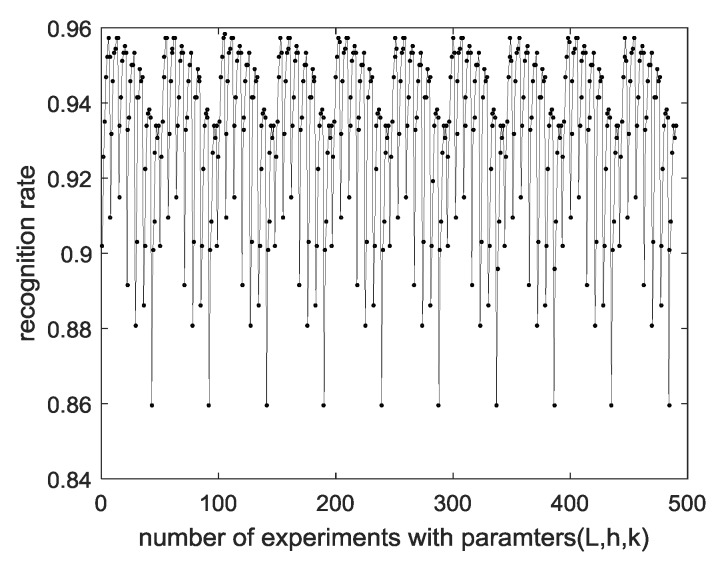
Performance of MIT-BIH database according to number of parameters.

In addition, we analyzed the results in terms of parameters of PCANet. To illustrate with visual data, Figure 17 is shown. As the number of L, which is a parameter of PCANet, increases, the performance of the recognition rate also increases. R did not significantly affect performance. Figure 17 fixes *R* and *h*, and increases *k* and *L* from 1 to 9. When *L* is 9, *k* is increased by 1, and when *k* is 9, *h* is increased by 1. It can be seen that 10 cycles are created by looking at the whole shape. This means that every time *L* becomes 9 and *k* becomes 9, a cycle occurs once every 50 times; as a result, the performance is affected by the parameters of *k* and *L*. In addition, when the lowest performance of 86% was shown, *L* was 3 and *k* was 9. That is, when *L* becomes *k* × *k*, the performance is low. 

On the other hand, we compared performance with existing algorithms. PCANet shows higher performance than PCA derived PCA. PCA showed stable performance between 30 and 40 eigenvectors. In addition, it shows better performance than AE, which is the basic model of deep running. The advantage of PCANet is that it is a simple deep-learning, but it is higher in performance than the existing deep learning. ELM is also a model that does not learn. That is, there is no backpropagation. ELM was not as high as PCANet’s performance despite its fast and popular algorithms. In the active function of the ELM, the sigmoid function, the relu function, and the sin function showed more than 85% performance. For the experiment, we designed the ensemble model using the ELM activation function, but the performance of PCANet was high. 

When analyzing DB, the performance of CU-ECG database was better than MIT ECG database. First, participants in the ECG CU database did not have heart disease, and, second, the CU-ECG database was the bigger than the MIT-BIH database. ECG is sensitive to heartbeat and heart disease. Therefore, the MIT-BIH database affects classification because of irregular data. In addition, the size of the data affected the learning of PCANet. The database of MIT-BIH is relatively small. 

On the other hand, we used the MIT-BIH ECG database in the field of recognition. The reason the MIT-BIH ECG database was used in the recognition field is that 47 patients did not judge the same class despite having the same disease (arrhythmia). Even in a few disease trials, arrhythmia, which has a significant effect on ECG, was not a major problem in ECG recognition, and the results are shown in Table 3. Figure 17 shows performance of MIT-BIH database according to number of parameters.

## 5. Conclusions

We herein propose the classification of ECG for identification systems using EECGNet from the human biosignal data. ECG authentication, which has been highlighted in the field of biometric signal authentication, was studied to design a simple and secure authentication system through a band-type clock. We designed the EECGNet-based SVM classifier for the ECG authentication system. The results show good performance when compared with other algorithms, and the validity of EECGNet was confirmed. The validity of EECGNet was confirmedto be 98.2% and the proposed method showed good performance when compared with conventional algorithms such as PCA, auto-encoder, ELM, and EELM. We verified the influence of these parameters on the performance of PCANet by modifying the key parameters of the experiment. Subsequently, we constructed an ECG dataset to perform the experiment and to verify whether the EECGNet can be used for the identification. In particular, EECGNet could influence the ECG certification system because of the advantage of the faster verification time. In the future, we plan to investigate EECGNet in three dimensions, as well as study the use of 1D ECG data and the structure change in the PCANet algorithm.

## Figures and Tables

**Table 1:** Core parameters of PCANet.

Parameters	Definition
m×n	The size of input image
*N*	The number of data
$k_{1}\times k_{2}$	The patch size. $k_{1}\;\mathrm{and}\;k_{2}$ are odd integers and satisfy $1\leq k_{1}\leq m,\;1\leq k_{2}\leq n.$
$L_{1}L_{2}$	The number of filters of two stages. $1\leq L_{1}\leq k_{1}k_{2},\;1\leq L_{2}\leq k_{1}k_{2}$
$h_{1}h_{2}$	The block size: 1$\leq h_{1}\leq m,\;1\leq h_{2}\leq n.\;Constraint:$ $h_{2}=\left[nh_{1}/m\right]$
R	The overlap ratio of block. R∈{0:0.1:0.9} which means R varies from 0 to 0.9 with the interval 0.1

**Table 2:** Performance of EECGNet using CU-ECG database.

h	K	L	Acc (*R* = 0.5)	Acc (*R* = 0.6)	h	K	L	Acc (*R* = 0.5)	Acc (*R* = 0.6)	h	K	L	Acc (*R* = 0.5)	Acc (*R* = 0.6)	h	K	L	Acc (*R* = 0.5)	Acc (*R* = 0.6)
6	3	3	92.68	94.89	8	3	3	92.68	92.78	10	3	3	92.68	92.68	12	3	3	92.68	92.68
	3	4	95.50	95.52		3	4	95.50	95.58		3	4	95.50	95.50		3	4	95.50	95.50
	3	5	97.35	97.37		3	5	97.35	97.38		3	5	97.35	97.35		3	5	97.35	97.35
	3	6	97.26	97.27		3	6	97.26	97.46		3	6	97.26	97.26		3	6	97.26	97.26
	3	7	96.98	96.99		3	7	96.98	96.99		3	7	96.98	96.98		3	7	96.98	96.98
	3	8	96.82	96.92		3	8	96.82	96.88		3	8	96.82	96.82		3	8	92.82	96.82
	3	9	96.77	96.97		3	9	96.61	96.79		3	9	96.67	96.77		3	9	96.77	96.77
	4	3	94.46	94.66		4	3	94.46	94.48		4	3	94.46	94.46		4	3	94.46	94.46
	4	4	96.39	96.79		4	4	96.39	96.49		4	4	96.39	96.39		4	4	96.39	96.39
	4	5	97.27	97.47		4	5	97.27	97.67		4	5	97.27	97.27		4	5	97.27	97.27
	4	6	97.74	97.84		4	6	97.74	97.84		4	6	97.74	97.84		4	6	97.74	97.94
	4	7	98.19	98.69		4	7	98.19	98.59		4	7	98.19	98.69		4	7	98.19	98.89
	4	8	98.00	98.20		4	8	98.00	98.20		4	8	98.00	98.50		4	8	98.00	98.50
	4	9	98.09	98.59		4	9	98.09	98.39		4	9	98.09	98.39		4	9	98.09	98.79
	5	3	95.52	95.62		5	3	95.52	95.82		5	3	95.52	95.62		5	3	95.52	95.92
	5	4	96.42	96.72		5	4	96.42	96.62		5	4	96.12	96.62		5	4	96.42	96.72
	5	5	97.20	97.60		5	5	97.20	97.40		5	5	97.20	97.50		5	5	97.20	97.50
	5	6	97.77	97.87		5	6	97.77	97.97		5	6	97.77	97.87		5	6	97.77	97.97
	5	7	97.95	97.98		5	7	97.95	97.99		5	7	97.95	98.15		5	7	97.95	98.15
	5	8	98.14	98.16		5	8	98.14	98.15		5	8	98.14	98.34		5	8	98.14	98.64
	5	9	98.21	98.28		5	9	98.21	98.24		5	9	98.21	98.31		5	9	98.21	98.71
	6	3	94.80	94.88		6	3	94.80	94.88		6	3	94.80	95.30		6	3	94.80	94.80
	6	4	96.78	96.79		6	4	96.78	96.79		6	4	96.78	97.28		6	4	96.78	96.98
	6	5	97.31	97.61		6	5	97.31	97.35		6	5	97.31	98.11		6	5	97.31	97.71
	6	6	97.43	97.63		6	6	97.43	97.63		6	6	97.43	97.63		6	6	97.43	97.43
	6	7	98.02	98.72		6	7	98.02	98.72		6	7	98.02	98.32		6	7	98.02	98.05
	6	8	98.26	98.36		6	8	98.02	98.56		6	8	98.02	98.56		6	8	98.26	98.27
	6	9	93.30	93.41		6	9	98.26	93.60		6	9	98.26	93.40		6	9	93.30	93.39
	7	3	96.08	96.28		7	3	93.30	96.88		7	3	93.30	96.58		7	3	96.08	96.58
	7	4	97.19	97.69		7	4	96.08	97.49		7	4	96.08	97.39		7	4	97.19	97.39
	7	5	97.68	97.78		7	5	97.19	97.88		7	5	97.19	97.78		7	5	97.68	97.78
	7	6	97.95	97.96		7	6	97.68	98.15		7	6	97.68	98.15		7	6	97.95	97.98
	7	7	97.96	97.98		7	7	97.95	97.99		7	7	97.95	97.99		7	7	97.96	9799
	7	8	98.01	98.06		7	8	97.96	98.09		7	8	97.96	98.05		7	8	98.01	98.05
	7	9	92.97	92.99		7	9	98.01	92.99		7	9	98.01	92.99		7	9	92.97	92.99
	8	3	95.11	95.21		8	3	92.97	95.61		8	3	92.97	95.16		8	3	95.11	95.12
	8	4	96.62	96.72		8	4	95.11	96.82		8	4	95.11	96.68		8	4	96.62	96.72
	8	5	97.08	97.88		8	5	96.62	97.58		8	5	96.62	97.38		8	5	97.08	97.78
	8	6	97.57	97.77		8	6	97.08	97.87		8	6	97.08	97.67		8	6	97.57	97.58
	8	7	97.65	97.85		8	7	97.57	98.15		8	7	97.57	97.68		8	7	97.65	97.68
	8	8	97.86	97.96		8	8	97.65	97.86		8	8	97.65	97.96		8	8	97.86	97.89
	8	9	92.18	92.38		8	9	97.86	92.98		8	9	97.86	92.38		8	9	92.18	92.19
	9	3	94.23	94.53		9	3	92.18	94.63		9	3	92.18	94.26		9	3	94.23	94.29
	9	4	96.74	96.84		9	4	94.23	96.94		9	4	94.23	96.79		9	4	96.74	96.79
	9	5	96.98	96.99		9	5	96.74	97.68		9	5	94.74	97.98		9	5	96.98	96.99
	9	6	91.57	91.77		9	6	96.98	91.67		9	6	96.98	91.47		9	6	91.57	91.67
	9	7	97.46	98.11		9	7	97.46	97.66		9	7	97.46	97.66		9	7	97.46	97.76
	9	8	97.54	97.58		9	8	97.46	97.84		9	8	97.46	97.58		9	8	97.54	97.84
	9	9	97.54	98.14		9	9	97.54	97.74		9	9	97.54	97.84		9	9	97.54	97.94

**Table 3:** Performance of EECGNet using MIT-BIH ECG database.

h	K	L	Acc (*R* = 0.5)	Acc (*R* = 0.6)	h	K	L	Acc (*R* = 0.5)	Acc (*R* = 0.6)	h	K		Acc (*R* = 0.5)	Acc (*R* = 0.6)	h	K	L	Acc (*R* = 0.5)	Acc (*R* = 0.6)
6	3	3	91.60	92.23	8	3	3	90.00	91.06	10	3	3	88.51	88.94	12	3	3	86.49	88.72
		3	4	92.98	93.19		3	4	93.09	93.30		3	4	92.23	92.87		3	4	90.21	92.77
		3	5	94.57	94.15		3	5	93.62	94.47		3	5	93.93	94.57		3	5	93.40	94.15
		3	6	94.68	94.89		3	6	94.89	95.00		3	6	94.36	94.68		3	6	94.47	95.00
		3	7	95.00	95.43		3	7	95.53	95.74		3	7	95.21	95.85		3	7	95.43	95.53
		3	8	95.53	95.64		3	8	95.11	95.43		3	8	95.64	95.85		3	8	95.21	95.85
		3	9	95.32	95.43		3	9	95.11	95.21		3	9	95.21	95.53		3	9	95.96	95.43
		4	3	92.34	92.34		4	3	90.96	91.06		4	3	88.62	89.68		4	3	86.60	89.15
		4	4	94.26	92.93		4	4	93.51	93.72		4	4	92.66	92.77		4	4	90.85	92.13
		4	5	95.00	95.11		4	5	94.26	94.89		4	5	94.15	94.57		4	5	93.94	94.26
		4	6	95.32	95.64		4	6	94.89	95.43		4	6	95.00	95.11		4	6	94.68	95.00
		4	7	95.32	95.43		4	7	95.53	95.32		4	7	95.64	95.53		4	7	95.43	95.64
		4	8	95.85	95.74		4	8	95.85	95.85		4	8	96.49	96.17		4	8	96.28	96.38
		4	9	95.74	95.53		4	9	95.64.	96.06		4	9	96.17	95.96		4	9	96.06	95.96
		5	3	93.30	92.98		5	3	92.55	92.66		5	3	89.47	91.70		5	3	88.09	89.68
		5	4	93.51	93.62		5	4	92.66	93.51		5	4	92.66	93.51		5	4	92.13	92.66
		5	5	94.68	94.57		5	5	94.79	94.36		5	5	94.15	94.47		5	5	94.79	94.47
		5	6	95.53	94.89		5	6	95.32	95.21		5	6	95.32	95.53		5	6	95.32	95.21
		5	7	95.32	95.53		5	7	95.64	95.32		5	7	95.21	95.43		5	7	95.85	95.21
		5	8	95.53	95.43		5	8	95.85	95.53		5	8	95.64	95.64		5	8	96.17	96.06
		5	9	95.64	95.32		5	9	95.74	95.11		5	9	95.64	95.53		5	9	95.53	95.43
		6	3	89.36	89.89		6	3	89.47	89.68		6	3	87.34	89.47		6	3	85.85	88.51
		6	4	93.09	93.72		6	4	93.30	93.30		6	4	91.70	92.77		6	4	90.53	92.13
		6	5	93.09	93.83		6	5	93.51	94.04		6	5	93.51	93.83		6	5	92.77	94.36
		6	6	94.79	95.21		6	6	95.21	95.00		6	6	94.79	95.32		6	6	94.57	95.21
		6	7	95.43	95.32		6	7	95.11	95.32		6	7	95.64	95.3		6	7	95.21	95.53
		6	8	94.89	94.89		6	8	95.21	95.21		6	8	95.74	95.53		6	8	95.64	96.06
		6	9	95.21	95.53		6	9	95.64	95.64		6	9	95.64	95.96		6	9	95.43	95.74
		7	3	89.04	89.26		7	3	87.13	88.72		7	3	87.23	87.13		7	3	85.53	86.49
		7	4	91.28	91.49		7	4	91.17	90.96		7	4	90.53	90.96		7	4	89.79	91.06
		7	5	94.04	94.26		7	5	94.04	94.36		7	5	93.62	93.83		7	5	93.19	93.51
		7	6	94.36	94.15		7	6	94.36	94.04		7	6	93.40	93.72		7	6	93.51	93.83
		7	7	94.57	94.89		7	7	95.00	95.00		7	7	94.36	95.00		7	7	94.47	94.57
		7	8	94.26	94.57		7	8	95.11	95.43		7	8	95.21	95.11		7	8	95.64	95.43
		7	9	94.79	94.79		7	9	94.68	95.43		7	9	95.74	95.64		7	9	95.64	95.43
		8	3	88.40	88.83		8	3	87.98	88.19		8	3	86.91	86.91		8	3	85.00	86.70
		8	4	90.11	90.32		8	4	89.57	89.04		8	4	88.62	88.72		8	4	87.87	88.51
		8	5	92.02	92.34		8	5	91.49	91.49		8	5	90.53	90.96		8	5	90.21	91.60
		8	6	93.40	93.40		8	6	93.51	93.40		8	6	93.09	93.19		8	6	92.02	93.40
		8	7	93.72	93.40		8	7	93.19	92.87		8	7	92.98	93.30		8	7	92.87	93.09
		8	8	93.72	93.83		8	8	93.94	92.83		8	8	93.83	94.04		8	8	93.83	94.15
		8	9	93.72	93.51		8	9	93.72	92.83		8	9	93.94	93.72		8	9	93.94	94.57
		9	3	86.06	87.23		9	3	85.96	85.43		9	3	84.57	85.32		9	3	83.19	84.47
		9	4	90.00	90.00		9	4	90.53	89.15		9	4	88.19	88.62		9	4	87.34	88.19
		9	5	91.06	80.85		9	5	90.64	91.06		9	5	90.74	90.96		9	5	90.32	90.74
		9	6	92.98	92.55		9	6	92.23	92.23		9	6	92.34	91.91		9	6	91.81	92.55
		9	7	93.30	93.40		9	7	93.72	92.87		9	7	93.19	93.09		9	7	92.98	92.98
		9	8	93.09	93.51		9	8	93.19	93.19		9	8	92.66	93.09		9	8	93.09	93.51
		9	9	93.62	93.94		9	9	95.51	93.51		9	9	93.94	93.62		9	9	93.72	94.04

**Table 4:** Comparison of EECGNet, PCA, AE, ELM, and EELM.

	Algorithm	PCA	ELM	EELM	AE	EECGNet
Database						
MIT-BIH ECG database		90.82%	85.72%	87.30%	91.25%	96.06%
CU-ECG database		96.45%	89.89%	91.24%	93.24%	98.87%
